# Identification of Genetic and Epigenetic Marks Involved in Population Structure

**DOI:** 10.1371/journal.pone.0013209

**Published:** 2010-10-07

**Authors:** Jingyu Liu, Kent Hutchison, Nora Perrone-Bizzozero, Marilee Morgan, Jing Sui, Vince Calhoun

**Affiliations:** 1 The Mind Research Network, Albuquerque, New Mexico, United States of America; 2 Department of Electrical and Computer Engineering, University of New Mexico, Albuquerque, New Mexico, United States of America; 3 Department of Psychology, University of New Mexico, Albuquerque, New Mexico, United States of America; 4 Department of Neurosciences, University of New Mexico, Albuquerque, New Mexico, United States of America; King Abdullah University of Science and Technology, Saudi Arabia

## Abstract

Population structure is well known as a prevalent and important factor in genetic studies, but its relevance in epigenetics is unclear. Very little is known about the affected epigenetic markers and their connections with genetics. In this study we assessed the impact of population diversity on genome wide single nucleotide polymorphisms (SNPs) and DNA methylation levels in 196 participants from five ethnic groups, using principle and independent component analyses. Three population stratification factors (PSFs) were identified in the genomic SNP dataset, accounting for a relatively large portion of total variance (6%). In contrast, only one PSF was identified in genomic methylation dataset accounting for 0.2% of total variance. This methylation PSF, however, was significantly correlated with the largest SNP PSF (r = 0.72, p<1E-23). We then investigated the top contributing markers in these two linked PSFs. The SNP PSF predominantly consists of 8 SNPs from three genes, *SLC45A2*, *HERC2* and *CTNNA2*, known to encode skin/hair/eye color. The methylation PSF includes 48 methylated sites in 44 genes coding for basic molecular functions, including transcription regulation, DNA binding, cytokine, and transferase activity. Among them, 8 sites are either hypo- or hyper-methylated correlating to minor alleles of SNPs in the SNP PSF. We found that the genes in SNP and methylation PSFs share common biological processes including sexual/multicellular organism reproduction, cell-cell signaling and cytoskeleton organization. We further investigated the transcription regulatory network operating at these genes and identified that most of genes closely interact with *ID2*, which encodes for a helix-loop-helix inhibitor of DNA binding. Overall, our results show a significant correlation between genetic and epigenetic population stratification, and suggest that the interrelationship between genetic and epigenetic population structure is mediated via complex multiple gene interactions in shared biological processes, through possibly, SNP-dependent modulation and *ID2* repressor function.

## Introduction

Genetic association studies analyze the connection between phenotypes (e.g. symptom of disease) and genotypes (e.g. single nucleotide variation) to identify genetic effects on the prevalence of diseases or other traits of interest. Such a study usually investigates large samples [1], [2], possibly from more than one population [3], [4], [5]. Additionally, the availability of genome wide genotyping has led to a rapid increase in the number of genome wide association studies (GWAS), which can analyze simultaneously over a million genetic loci in one study. The confounding effect of population diversity on allelic frequencies may alter the results and inflate false positives [6], [7], [8]. Thus it is critical to understand the population structure in a given sample set and account for it before performing association analyses with other factors.

A similar situation holds true for epigenetic studies, i.e. studies of heritable phenomena that regulate gene expression and hence genetic function without altering the DNA sequence. Epigenetic inheritance has been documented in a substantial number of animal studies and human studies [9], [10], [11], [12]. DNA methylation, one of the epigenetic mechanisms, is essential for normal cellular differentiation, parental imprinting and X inactivation, and is also prone to perturbation by environmental factors. A number of DNA methylation studies have identified methylation changes with diet, smoking, drinking, age, gender and lifestyle [13], [14], [15], [16], [17], [18]. DNA methylation changes are also associated with carcinogenesis where genome-wide hypomethylation and some gene specific hypermethylation were observed in cancer cells [9]. However, the relationship between the diversity of population and the diversity of epigenetic marks is poorly understood. Only a couple of studies have demonstrated ethnic difference in the overall DNA methylation level [19] or methylation of some specific genes involved in cancer [20]. Furthermore, Nielsen et al. have observed the ethnic diversity of *OPRM1* gene methylation in heroin addicts [21]. Ultimately, the identification of the population structure embedded in epigenetic data will be critical for understanding the epigenetic influence on disease processes and the interaction between epigenetics and environmental factors.

Additionally, the relationship between genetic and epigenetic marks in terms of population structure remains unknown. To address this issue in this study we sought to answer the following questions: Does a similar population structure exists in genetic and epigenetic datasets? Do the same genes or biological pathways contribute to population structure? From a population stratification perspective, how strongly are epigenetic and genetic marks correlated? When genome-wide SNP genotypes and DNA methylation are obtained from the same participants, we are given the opportunity to investigate these questions directly.

In this study, DNAs derived from saliva DNA of 196 participants were analyzed in Illumina Human 1M-Duo Single Nucleotide Polymorphism (SNP) arrays and Illumina Infinium 27K methylation arrays. Based on self report ethnicity, the participants are comprised of Caucasian, Latino, African American, Native American, Asian and mixed group. We first examined the population structure in the SNP array and methylation data separately, and then studied the inter-relation between them.

## Results

### 1). SNP population stratification factors (PSF)

**Figure 1:**
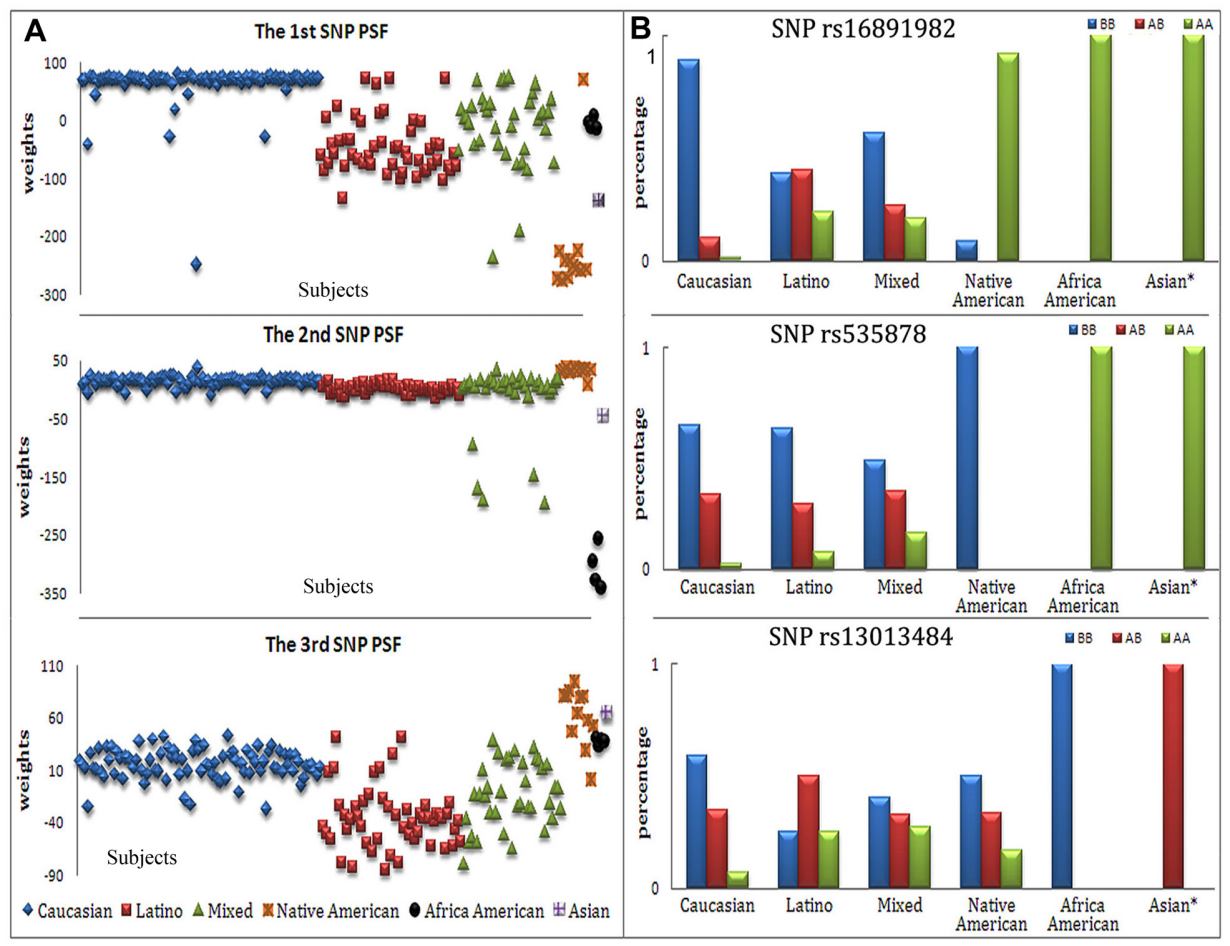
Stratification of the population using SNP PSFs. a) Relative weights of three SNP PSFs in the subjects. X axis represents the individual subjects, while Y axis shows the SNP weights. b) The top SNP's allelic distribution. Note: there is only one Asian subject.

A total of 195 principle component (PC) factors were extracted from genomic SNP array and 3 of them were identified as PSFs by correlation with ethnicity, which are the 1st, 2nd, and 6th PCs. These three PCs account for 6% of genome wide SNP variance, with the 1st one alone accounting for 4%. ANOVA test results in Table 1 show how each PSF differs in ethnic groups, indicated by the p value of group difference and percentage variance explained by each ethnic group. Based on a |Z|>4 threshold, the top contributing SNPs for each PSF are also listed; eight SNP loci are identified for the 1st PSF, 12 SNP loci for the 2nd PSF, and 15 SNP loci for the 3rd PSF (genes associated are provided in the supporting Table S1). To present more clearly the population structure in the three PSFs, we plotted the distributions of relative weights of the PSFs in all ethnic groups, as well as the top SNP genotype distribution in the population (Figure 1). Relative weights were projection values calculated in a factorization method, showing how the data present in a particular direction. Here the direction is the PSF derived from linear combination of SNPs genotype, and weights show how the PSF reveals itself in each subject The 1st PSF is able to differentiate Caucasian, Latino and Native American groups, but it works better for Caucasians and Native Americans, each explaining about 40% of the PSF variance. The top SNP rs16891982 (*SLC45A2*) demonstrates a varying allelic frequency from Caucasian to African American, where Caucasians have a marked high frequency of genotype BB (i.e. homozygous for major allele G), Native Americans and African Americans have marked high frequencies of genotype AA (i.e. homozygous for minor allele C), and Latinos have approximately same frequency of genotype BB and AB. The 2nd PSF only differentiates African Americans from others with African America group explaining 68% of this PSF variance. SNP rs535878 (*MOBP*) shows that only African Americans have a remarkable high frequency of genotype AA (allele C). The 3rd PSF can differentiate Caucasian, Latino and Native America, but mostly for Latino, which explains 36% of this PSF variance. SNP rs13013484 shows that all ethnic groups excluding Asian and Latinos, express the highest frequency of genotype BB (allele A), whereas the most frequent genotype in Latinos is AB, with the same rate for allele A and G.

**Table 1:** PSFs and their differences in ethnic groups.

PC/IC Number	Group difference*P value & Variance explained (η^2^)	Top contributing sites
SNP: Top contributing SNPs (|Z|>4)		
1st	Caucasian: p<1E-23; η^2^ = 0.40, Latino: p<1.61E-5; η^2^ = 0.09, Native American: p<1E-23; η^2^ = 0.41.	rs16891982, rs35389, rs35407, rs35391, rs35412, rs28117, rs3755095, rs12913832
2nd	Caucasian: p<6E-4; η^2^ = 0.06, African American: p<1E-23; η^2^ = 0.68.	rs535878, rs326946, rs594624, rs11709533, rs6809442, rs1708000, rs1606473, rs2909679, rs13066103, rs4725537, rs11129837, rs16891982
6th	Caucasian: p<1.16E-8; η^2^ = 0.15, Latino: p<1.61E-5; η^2^ = 0.36, Native American:p<4.41E-11; η^2^ = 0.20.	rs13013484, rs9677663, rs9967838, rs6080826, rs13075278, rs3792252, rs3736594, rs4814656, rs10829792, rs11708297, rs10762401, rs1966963, rs334543, rs12147353, rs2150392
Methylation: Top contributing genes (|Z|>6)		
22nd	Caucasian: p<1E-23; η^2^ = 0.32, Latino: p<4.69E-5; η^2^ = 0.08, Native America: p<5.55E-16; η^2^ = 0.29.	48 sites in 44 genes from 18 chromosomes

*Results are derived from ANOVA analyses of one group again all others.

### 2). Methylation PSF

**Figure 2:**
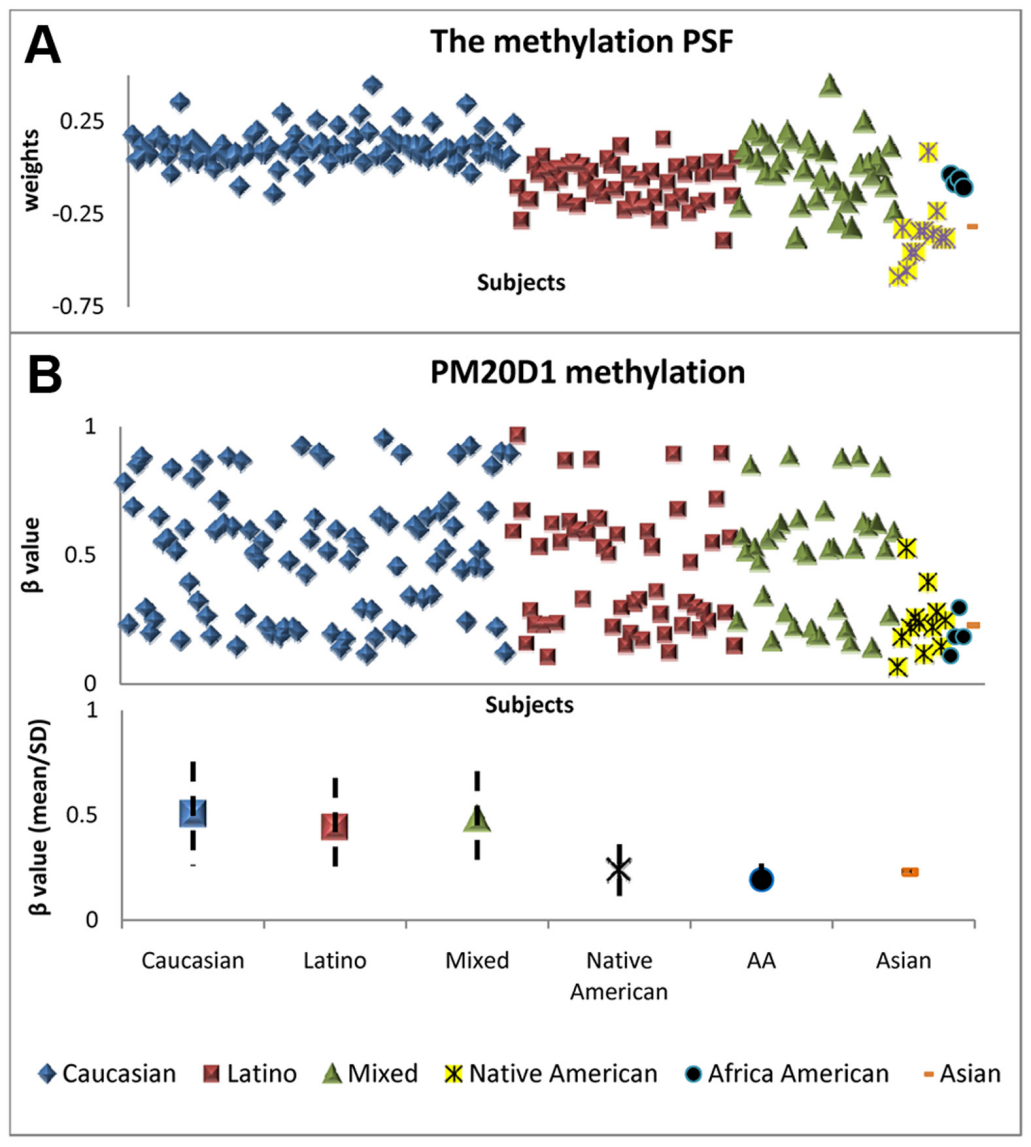
Distribution of the methylation PSF in population. a) Relative weights of the PSF in the subjects. X axis represents the individual subjects, while Y axis shows the SNP weights b) *PM20D1* methylation β values in the subjects sorted by ethnic groups. The mean and standard deviation of *PM20D1* methylation values for each ethnic group are also plotted, with no standard deviation for the one Asian subject.

A total of 35 independent components (ICs) were extracted by independent component analysis in methylation data and only one, the 22th IC was identified as a methylation PSF, accounting for 0.2% of total genomic methylation variance. In this PSF, as reported in Table 1, three groups, Caucasian, Latino and Native American show significant differences, and altogether they explain 69% of variance of the PSF. The top 48 methylated sites (|Z|>6) in 44 genes, 18 Chromosomes are selected as the prominent contributing sites for this PSF (see Supporting Table S2 for the details). Figure 2A presents this PSF relative expression weight per subject in each of the 5 groups. Caucasians show a relative high expression, then Latinos, followed by African Americans and Native Americans. Among the 48 sites, some show methylation patterns similarly to the PSF, as exemplified in Figure 2B using methylation β values of the PM20D1 gene. The mean and standard deviation (SD) of methylation levels in each ethnic group is also plotted, which shows that Caucasians present the highest methylation level, followed closely by Latinos and the mixed group, while native Americans and African Americans have similar low methylation levels. Others show methylation patterns with an opposite-direction population structure, negatively contributing to the PSF. Twenty three of these PSF methylation sites are in CpG islands, resulting a 48% CpG island rate. Compared with the overall 64% CpG island rate in the methylation data, the PSF methylation sites tends to have a lower probability of being located in CpG islands.

### 3). Relationship between genetic and epigenetic PSFs

**Figure 3:**
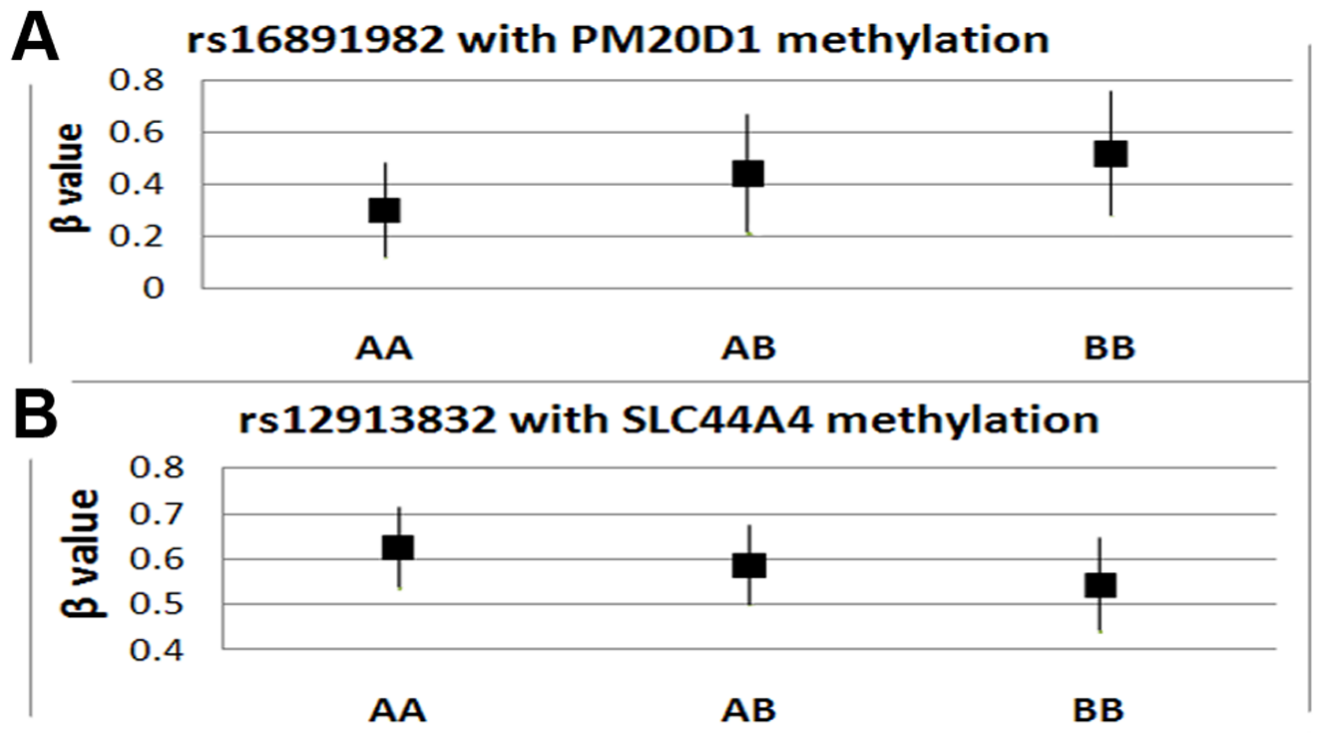
The association of SNPs and DNA methylation. a) rs16891982 positively correlates with the methylation level of *PM20D1*; minor allele A is associated with lower methylation β values of *PM20D1*. b) rs12913832 negatively correlates with the methylation level of *SLC44A4*, i.e. minor allele A is associated with higher methylation β values of *SLC44A4*.

Pair wise correlation tests between SNP PSFs and the methylation PSF show that only one pair of PSFs, the first SNP PSF and the methylation PSF, are significantly associated with a correlation coefficient R of 0.72 (p value<1E-23). We further analyzed the top contributing sites in the linked PSFs. Of the top 8 SNPs, rs16891982, rs35389, rs35407, rs35391, rs35412 and rs28117 are in the *SLC45A2* gene (the solute carrier family 45, member 2 encoding a transporter protein that mediates melanin synthesis). SNP rs3755095 is in *CTNNA2* (encoding alpha 2 catenin). SNP rs12913832 is in *HERC2* (encoding the HECT domain and RCC1-like domain-containing protein 22). The top 48 methylation sites are in 44 genes covering a broad spectrum of biological/molecular functions. To better understand the potential association of SNPs on methylation levels, we tested the correlation between the 8 SNPs' genotypes and the 48 methylation sites. After Bonferroni correction (P value<4E-4), 8 methylation sites were significantly correlated with the SNPs listed in Table 2. A positive correlation implies that the minor allele of a SNP is associated with hypomethylation of a methylated site, while negative correlation indicates a hypermethylation association. All 8 SNPs are significantly associated with the methylation of one or more methylated sites. Two significant associations (one positive, one negative) are shown in Figure 3. SNP rs16891982 (along with all other 5 SNPs in *SLC45A2*, not plotted) shows a significant positive correlation with the methylation β values of *PM20D1*; i.e. the minor allele is associated with lower β values of *PM20D1*. Besides *PM20D1*, SNP rs16891982 also correlates with methylation of *CDC42BPA*, *IL6* and *SERPINB3*, but negatively. In Figure 3B, SNP rs12913832 in *HERC2* shows a significant negative correlation with *SLC44A4* methylation. Moreover, rs12913832 is also negatively associated with methylation of *CDC42BPA*, *FAM181A* and *SERPINB3*, and positively associated with *HLA-DRA* (not plotted but listed in Table 2). To further ensure the associations of SNPs and methylation level are real, we tested the top 8 SNPs from all 195 SNP principle components. From a total of 1552 SNPs excluding the 8 SNPs we identified, 5% were correlated with the methylation of PSF methylated sites, reflecting the 5% false positive control.

**Table 2:** Methylation sites associated with the PSF SNPs.

Methylation sites	SNPs (genes)	Relation	Association involved functional clusters
*PM20D1*	all 8 SNPs from genes *SLC45A2*, *HERC2* and *CTNNA2*	positive	zinc-finger/zinc ion binding/transition metal ion binding; cation/ion/metal/metal ion binding
*SLC44A4*	rs3755095(*CTNNA2*), rs12913832 (*HERC2*)	negative	
*CDC42BPA*	rs16891982, rs35389, rs35407, rs35391, rs35412, rs28117(*SLC45A2*) and rs12913832(*HERC2*)	negative	zinc-finger/zinc ion binding/transition metal ion binding; cation/ion/metal/metal ion binding
*IL6*	rs16891982, rs35389, rs35407, rs35391 (*SLC45A2*) and rs3755095(*CTNNA2*)	negative	cell-cell signaling
*HLA-DRA*	rs12913832(*HERC2*)	positive	
*STK38*	rs3755095(*CTNNA2*)	positive	
*FAM181A*	rs12913832(*HERC2*)	negative	
*SERPINB3*	rs16891982(*SLC45A2*), rs12913832(*HERC2*)	negative	

Of the total 47 genes from both SNP and methylation PSFs, we identified 13 shared functional clusters based on functional categories, gene ontology (GO) and pathway information. These 13 clusters (see Supporting Table S3) focus on functions of, 1) taxis/chemokine/cytokine activity with emphasis on inflammatory/immune/wound/defense response, 2) extracellular matrix, 3) actin cytoskeleton organization, 4) sexual reproduction and multicellular organization reproduction, 5) cell morphogenesis and cellular component morphogenesis, 6) macromolecule catabolic process and protein catabolic process, 7) zinc-finger, zinc ion binding, 8) phosphorus metabolic process and intracellular signaling cascade, 9) cation/ion/metal ion binding, 10) purine ribonucleotide/adenyl ribonucleotide/ATP binding, 11) intracellular organelle lumen, organelle lumen and nuclear lumen, 12) transmembrane region, intrinsic to member and integral to membrane, and 13) transcription regulation. Importantly, 7 clusters (Clusters 1, 4–7, 9, 12) include biological processes/pathways where both SNP and methylation PSF genes are involved (these pathways are highlighted using * in the Supporting Table S3). Furthermore, clusters 1, 7 and 9 include processes in which direct associations of SNPs with hypo- or hyper-methylation were identified (see Table 2).

**Figure 4:**
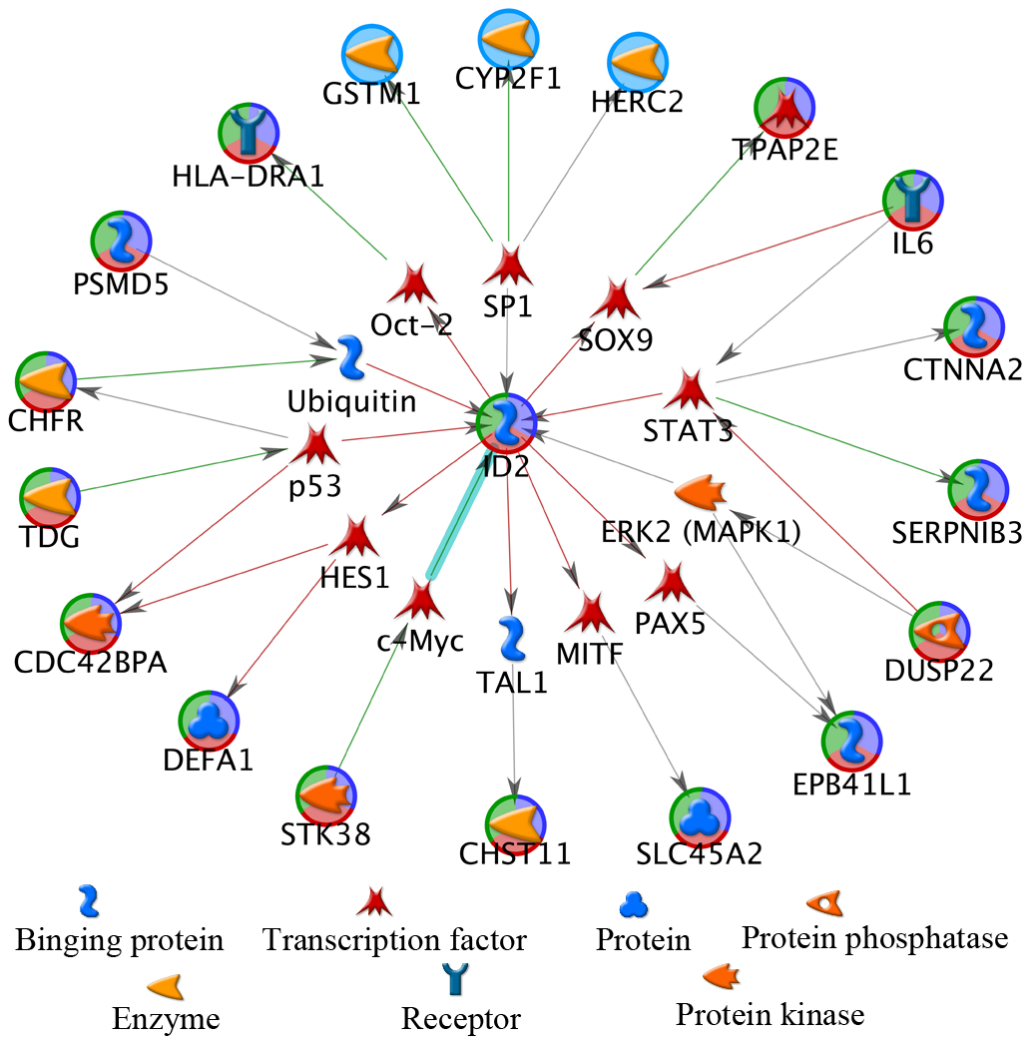
Biological network linking SNP PSF genes with methylation PSF genes. Nineteen gene products encoding various proteins are one node away from *ID2*, mostly connected via a transcription factor. Among these are the 3 genes in the SNP PSF (*HERC2*, *CTNNA2* and *SLC45A2*) and 16 genes in the methylation PSF.

To further investigate the relationship of the SNP and methylation PSFs, a network focusing on the transcription factors among all the 47 genes was extracted using the shortest path of 2 steps tool in MetaCore™ software. In order to represent the main network clearly, we centered the network at the *ID2* transcriptional repressor, removed the extended connections (2 steps away from *ID2*) (Figure 4). Nineteen genes in the SNP and methylation PSFs are connected to *ID2* through 1 node, frequently involving a transcription factor. Additional connections exist between these gene products, but, for display purposes, we only show those related to transcriptional regulation.

## Discussion

As expected for genomic SNP array data, clear population structure information was extracted, resulting in three PSFs, which are ranked as the 1st, 2nd, and 6th PC in a descending order of variance. These three PSFs accounted for a relatively large portion of total variance in the whole SNP array (6% of total 195 PCs), reinforcing the importance of defining population stratification in GWAS. The three PSFs comprise different SNPs, which differentiate each of the ethnic groups in a unique manner. The first PSF reveals positive weights in Caucasians, close to zero in Latinos and African Americans, and negative weights in Native Americans and Asians, with mixed group lying between Caucasians and Latinos. Considering the uncertainty of self reporting individual ethnicities, it is not surprising to see some participants showing expression deviating from their own ethnic group. Overall, this PSF allows separation in all ethnic groups. The 2nd and 3rd PSFs are more focused on one specific ethnic group, African American and Latino respectively. It is possible there are more PSFs that can be identified, but given we do not have a large subject sample size, we use a stringent criterion of p<1E-4.

Only one PSF was identified in methylation ranked as the 22nd IC, accounting for much less variance (0.2% of total methylation variance) compared to the PSFs identified in the SNPs, which implies that population structure does affect methylation but is not the largest influencing factor. This PSF shows decreasing weights from Caucasians to Latinos and African Americans, and then to Native Americans and Asians. This trend can also be seen in the example of gene *PM20D1* methylation (Figure 2B), though with large deviations. It again indicates that ethnicity affects methylation, but large portion of methylation variation is induced by other factors. Since epigenetics is prone to environmental influence, we are not surprised to see that ethnicity only plays a relatively small role in methylation variation.

Three genes, 8 SNP loci, contribute most to the 1st PSF in SNPs (we used a conservative empirical threshold to select the top contributing loci and hence might have missed SNPs contributing less to the PSF). *SLC45A2* encodes a transporter protein that is involved in melanin synthesis. Melanin serves predominantly as a pigment in humans, and is the primary determinant of skin, hair and eye color. This is consistent with the differences in skin pigmentation seen among different ethnic groups. *HERC2* belongs to the *HERC* (HECT domain and RCC-1 domain) gene family and it is thought to encode an E3 ubiquitin-ligase. Genetic variations in this gene has been reported to be associated with hair color, eye color and skin color [5], [22], [23] and type 1 diabetes [24]. In particular, the top SNP we identified in this study rs12913832 is repeatedly identified to determine eye color. [23], [25], [26]. *CTNNA2* encodes alpha N-catenin, a protein that links the cadherins adhesion receptor to the neuronal cytoskeleton and is expressed mainly in the nervous system. Animal studies show that *CTNNA2* is essential for the stability of dendritic spines and synaptic contacts [27], and also for normal cerebellar and hippocampal morphology [28]. However, there is no information about its connection with population structure as yet.

Forty-four genes are identified as the top contributing sites to the methylation PSF (Supporting Table S3). These 44 genes cover a broad spectrum of molecular functions in many biological processes. To the best of our knowledge, they encode proteins responsible for very basic functions such as DNA transcription and regulation (*ID2, LASS3, TFAP2E, KLF17, ZNF205*), DNA binding and protein binding (*TDG, ID2, ZNF205, DNAJB7*, *NLRP5*, *CDC42BPA, CSDC2, KLF17, MGC3207, PSMD5, STK38*), metal ion binding and calcium ion binding (*CAPN9, SUSD1, CHFR, CYP2F1, PM20D1*), hydrolase activity and metallopeptidase activity (*PM20D1, DUSP22D*), cytokine activity and chemokine activity (*CCL4, CCL26, IL6, CCL4L2*), and sulfotransferase activity and transferase activity (*PPP4R2, CHST11, SULT1C1, GSTM5, GSTM1, UGT2B17*). More clearly, the 13 overrepresented functional clusters confirm the main biological processes.

Though there is no overlap between genes contributing to the SNP PSF and methylation PSF, we did identify the association of SNPs in the SNP PSFs with the DNA methylation of sites in methylation PSF. Specifically, a remarkable similarity was found between the 1st SNP PSF and the methylation PSF manifesting as a correlation of 0.72 (P<1E-23), which strongly suggests the existence of a population structure linkage between genetics and epigenetics. As we show in two examples in Figure 3, the SNPs (rs16891982 and rs12913832), known to encode pigmentation in humans [23], [25], [29], appear to modulate the methylation level of other genes (*PM20D1* and *SLC44A4*). These modulations were further confirmed by testing all possible SNP components, where 5% of SNPs in general showed correction, yet 100% of the top 8 SNPs in the SNP PSF showed significant correlations. Given that there are no direct associations of the genes containing these 8 SNPs with any known DNA methylases, this implies that the genetic influence on the epigenetic population structure is more complex, possibly through interactions involving multiple genes. This implication can be further strengthened by the identified 7 functional pathway/biological process clusters, where both genetic and methylation PSF genes are involved. In particular, we found SNP to hypo- or hyper-methylation associations (Table 2), which participate three functional clusters. Finally, we identified a biological network with multiple-gene interactions, where most genes are closely connected to the *ID2* protein via a transcription factor. This protein belongs to the inhibitor of DNA binding (ID) family. Members of the ID family inhibit the functions of basic helix-loop-helix (bHLH) transcription factors in a dominant-negative manner by suppressing their heterodimerization partners through the HLH domains. Even though the detailed mechanism is unknown, *ID2*, known to down- or up- regulate gene expression in cell differentiation and proliferation, could potentially work as a master regulator corrdinating the expression of the genes in the SNP and methylation PSFs described in our study. The hypothesis of shared biological process bridging the population structure in genetics and epigenetics, suggested by our results, needs a detailed molecular level investigation.

In summary, remarkably similar population stratification patterns were discovered in the genetic and epigenetic data. While the bases for this are presently unclear, our results suggest that 1) the genetic population structure is determined by a small set of focused genes; 2) in contrast, multiple genes coding for basic molecular functions are regulated by the population structure to a small extent in DNA methylation data; 3) the interconnection between genetic and epigenetic PSFs is more likely achieved through complex multiple gene interactions involved in shared biological processes, possibly through specific SNP to methylation modulation, and which in turn could be regulated by *ID2*. Further in-depth studies are needed to test this hypothesis.

## Materials and Methods

### 1). Subjects

The study was conducted according to the principles expressed in the Declaration of Helsinki, approved by the Institutional Review Board of University of New Mexico. All participants provided written informed consent for the collection of samples and subsequent analysis. One hundred ninety-six participants including 53 females with age 32.21± 10.74 and 143 males with age 32.22± 9.70 were investigated in this study, which is a subsample of an on-going study designed to investigate genetic/epigenetic prediction for substance dependence. Subjects between age 21 and 55 (right handed) with a minimum alcohol consumption of a regular pattern of two binge drinking episodes per week, otherwise healthy (no maximum alcohol consumption limit, but no history of severe brain injury or brain related medical problems, no symptoms of psychosis during a diagnostic interview), were included.

The demographic information and alcohol dependence level were assessed through questionnaires. We gave the alcohol use disorder identification test (AUDIT) [30] to participants and obtained a score for alcohol dependence level. The self report ethnicity shows that all participants are comprised of five ethnic groups listed in Table 3. There is no significant (p<0.05) difference in age, gender, or AUDIT score between ethnic groups (no test was performed on the Asian group).

**Table 3:** Demographic information.

Ethnicity	Number	Gender (m/f)	Age	AUDIT
Caucasian	90	64/26	32.4+10.2	18.9+7.6
Latino	52	36/16	32.8+10.3	19.1+7.5
African American	4	4/0	34.0+5.0	21.8+6.1
Mixed	37	28/9	30.1+9.5	18.7+8.0
Native American	12	10/2	35.3+10.1	21.5+6.8
Asian	1	1/0	24	11

### 2). SNP Genotyping

Participants were instructed to deliver 5 ml of saliva into a sterile 50 ml conical centrifuge tube. DNA was then extracted, purified and ready for genotyping based on Illumina human 1M-duo SNP assay recommendation. 1,199,187 loci were genotyped. A quality control was set to select readings with a call rate above 99%. Loci with more than 5 missing values were excluded, otherwise filled using the most common genotype in the given dataset. We code the three genotypes, AA, AB and BB, as 1, 0, and -1 without assuming dominate or recessive model. Preprocessing of SNP array was preformed, including removing loci with minor allele frequency less than 0.01, redundant loci with correlation higher than 0.95, and loci in sex chromosomes. A total of 541,204 loci were included at the end for further analysis.

### 3). Methylation measurement

The same DNA extracted from saliva was used for methylation detection. First, DNA was bisulfite converted to separate methylated and unmethylated sites, and then PCR amplified and hybridized. The Illumina Infinium Methylation Assay was used to detect genome wide 27,578 CpG sites, spanning 14,495 genes. The CpG sites locate within the proximal promoter regions of genes, with distance to transcription start site ranging from 0 to 1499 bp averaged at 389±341 bp. A methylation β value was output for each site, which is a continuous variable between 0 and 1, representing the ratio of the intensity of the methylated type to the total intensity. Zero means no methylation, and one means 100% methylation. The reproducibility of Illumina assay methylation is reported as R2 of 0.98, and the standard deviation of methylation values from replicates is less than 0.06 [31]. Among the 27,578 CpG sites, some sites have shown either low level averaged methylation or low level variation among all 196 subjects. They thus convey very limited information for further study and great influence of measurement errors. We eliminated these sites using an empirical threshold setting of averaged methylation being 0.05, or variance being 0.0003. This results in 18,960 CpG sites from both autosomes and sex chromosomes. A gender effect correction was performed on methylation in both autosomes and sex chromosomes[16]. Then 17,966 autosomal sites were used for further study. 64% of methylation sites were located in CpG islands.

### 4). Analyses methods

4.1). Principle component analysis (PCA) is a data driven projection method. It uses variance information to project the original data into a new coordinate system, principle component system. All PCs are orthogonal to each other and present a certain amount of variance, ranked in a descending order. PCA has been proposed to analyze population stratification in a number of genetics studies [32], and has been implemented in several popular analysis tools such as PLINK [33] and EigenStrat [32]. Each PC extracted in the genetic data infers a continuous axis of genetic disparity. If the genetic disparity is significantly linked to ethnicity information, the PC will be recognized as a population stratification factor. Based on the factor's projection coefficients, we can also roughly identify the top contributing loci, those with higher coefficients than a threshold (an empirical |Z| score>4, explained later in detail). In this study we applied PCA on the SNP array to extract genetic population structure information. Among the maximum possible 196 components (limited by the original 196 dimensions/samples), 195 PCs extracted from PCA had non-zero variance.

***4.2).*** Independent component analysis (ICA) is also a data driven method. Similarly, it uses high order statistics of data to extract independent components, which satisfy more stringent criteria than orthogonality in PCA. Each independent component presents a particular source contributing to the observations (data) and is associated with a specific loading pattern indicating how the source is weighted and added into observations. If the loading pattern shows significant differences for different ethnic groups, then it will be considered as a PSF. We can further analyze each PSF source to identify the top contributing sites. In this study, we conducted ICA on the methylation data using Infomax algorithm [34]. The number of ICs to retain in the ICA on methylation data is estimated by maintaining 90% of the total variance to balance maximizing information and minimizing measurement noise. This resulted in thirty-five ICs being extracted.

From the factorization viewpoint, ICA can be categorized as an extension of PCA, using more strict (in some cases additional) criteria to refine the components. For some components (most likely the components capturing more variance), there is no obvious difference between ICA and PCA results. That is the case when we applied ICA and PCA on the SNP data in this study; similar PSF components were extracted. But to compare with the literature where PCA has been used to extract genetic population structures, we also applied PCA onto the SNPs. For methylation data ICA produced a stronger PSF component, likely due to the relatively weak population diversity reflected in methylation.

***4.3).*** ANOVA tests are performed for each PC or IC extracted from SNPs or methylation data separately, to examine the existence of ethnic group differences (except for Asian). The PC(IC) showing significant group differences (p value<1E-4) presents, to an extent, the population structure embedded in genetic or epigenetic data. Thus we termed the PC (IC) a PSF. For each PSF, we also calculated the percentage of variance explained by each ethnic group, which provides additional information about the population structure.

***4.4).*** Pearson correlation tests were performed on all pairs of SNP PSFs and methylation PSF. A significant correlation (passing multiple comparison correction) indicates the presence of interconnection between genetic and epigenetic population structure. We then identified the loci contributing to the linked PSFs, by Z transforming the PSFs' projection coefficient or source (removing the mean and dividing by the standard deviation), and selecting the loci with Z score higher than an empirical threshold (4 for SNPs, and 6 for methylation based on a subjectively observed break point in the Z score distribution). We subsequently correlated directly the genotypes of each identified contributing SNP with the β methylation values of each identified methylation site, and by doing so we were able to test the potential modulation function of SNP genotypes on methylation levels.

***4.5).*** To localize the shared biological functions or involved pathways of the contributing genes from both SNP and methylation PSFs, we used the gene functional annotation clustering tool, built into the database for annotation, visualization and integrated discovery (DAVID, http://david.abcc.ncifcrf.gov)[35], [36]. This tool identified clusters (similarity) of many gene functions. The clusters were extracted using default clustering criteria, including functional categories, gene ontology and pathway information, and the classification stringency was set as high**.** We further investigated biological networks among all the PSF genes focusing on transcription regulation factors, using MetaCore™ from GeneGo Inc. [http://www.genego.com/metacore]. The network was built via the shortest path of maximum 2 steps, and using only curated interactions and functional and binding interactions.

## Supporting Information

Table S1 8 SNPs in the 1st genetic PSF
(0.05 MB DOC)

Table S2 Forty-eight sites in the methylation PSF
(0.11 MB DOC)

Table S3 Biological process/annotation clusters
(0.07 MB DOC)

## References

[1] Wellcome-Trust-Case-Control-Consortium (2007). Genome-wide association study of 14,000 cases of seven common diseases and 3,000 shared controls.. Nature.

[2] Edenberg HJ, Foroud T (2006). The genetics of alcoholism: identifying specific genes through family studies.. Addict Biol.

[3] Potkin SG, Turner JA, Guffanti G, Lakatos A, Fallon JH (2009). A genome-wide association study of schizophrenia using brain activation as a quantitative phenotype.. Schizophr Bull.

[4] Liu XG, Tan LJ, Lei SF, Liu YJ, Shen H (2009). Genome-wide association and replication studies identified TRHR as an important gene for lean body mass.. Am J Hum Genet.

[5] Kayser M, Liu F, Janssens AC, Rivadeneira F, Lao O (2008). Three genome-wide association studies and a linkage analysis identify HERC2 as a human iris color gene.. Am J Hum Genet.

[6] Yu K, Wang Z, Li Q, Wacholder S, Hunter DJ (2008). Population substructure and control selection in genome-wide association studies.. PLoS ONE.

[7] Wang K (2009). Testing for genetic association in the presence of population stratification in genome-wide association studies.. Genet Epidemiol.

[8] Patterson N, Price AL, Reich D (2006). Population structure and eigenanalysis.. PLoS Genet.

[9] Tost J, Tost J (2008). DNA Methylation: An Introduction to the Biology and the Disease-Associated Changes of a Promising Biomarker.. DNA Methylation: Methods and Protocols.

[10] Chandler VL (2007). Paramutation: from maize to mice.. Cell.

[11] Pembrey ME, Bygren LO, Kaati G, Edvinsson S, Northstone K (2006). Sex-specific, male-line transgenerational responses in humans.. Eur J Hum Genet.

[12] Kaati G, Bygren LO, Pembrey M, Sjostrom M (2007). Transgenerational response to nutrition, early life circumstances and longevity.. Eur J Hum Genet.

[13] Sutherland JE, Costa M (2003). Epigenetics and the environment.. Ann N Y Acad Sci.

[14] Jacob RA, Gretz DM, Taylor PC, James SJ, Pogribny IP (1998). Moderate folate depletion increases plasma homocysteine and decreases lymphocyte DNA methylation in postmenopausal women.. J Nutr.

[15] Chang HW, Ling GS, Wei WI, Yuen AP (2004). Smoking and drinking can induce p15 methylation in the upper aerodigestive tract of healthy individuals and patients with head and neck squamous cell carcinoma.. Cancer.

[16] Liu J, Morgan M, Hutchison K, Calhoun V (2010). A Study of the Influence of Sex on Genome Wide Methylation..

[17] Brait M, Ford JG, Papaiahgari S, Garza MA, Lee JI (2009). Association between Lifestyle Factors and CpG Island Methylation in a Cancer-Free Population.. Cancer Epidemiol Biomarkers Prev.

[18] Shukla SD, Velazquez J, French SW, Lu SC, Ticku MK (2008). Emerging role of epigenetics in the actions of alcohol.. Alcohol Clin Exp Res.

[19] Terry MB, Ferris JS, Pilsner R, Flom JD, Tehranifar P (2008). Genomic DNA methylation among women in a multiethnic New York City birth cohort.. Cancer Epidemiol Biomarkers Prev.

[20] Das PM, Ramachandran K, Vanwert J, Ferdinand L, Gopisetty G (2006). Methylation mediated silencing of TMS1/ASC gene in prostate cancer.. Mol Cancer.

[21] Nielsen DA, Hamon S, Yuferov V, Jackson C, Ho A Ethnic diversity of DNA methylation in the OPRM1 promoter region in lymphocytes of heroin addicts.. Hum Genet.

[22] Han J, Kraft P, Nan H, Guo Q, Chen C (2008). A genome-wide association study identifies novel alleles associated with hair color and skin pigmentation.. PLoS Genet.

[23] Branicki W, Brudnik U, Wojas-Pelc A (2009). Interactions between HERC2, OCA2 and MC1R may influence human pigmentation phenotype.. Ann Hum Genet.

[24] Wang K, Baldassano R, Zhang H, Qu HQ, Imielinski M Comparative genetic analysis of inflammatory bowel disease and type 1 diabetes implicates multiple loci with opposite effects.. Hum Mol Genet.

[25] Sturm RA, Duffy DL, Zhao ZZ, Leite FPN, Stark MS (2008). A single SNP in an evolutionary conserved region within intron 86 of the HERC2 gene determines human blue-brown eye color.. American Journal of Human Genetics.

[26] Eiberg H, Troelsen J, Nielsen M, Mikkelsen A, Mengel-From J (2008). Blue eye color in humans may be caused by a perfectly associated founder mutation in a regulatory element located within the HERC2 gene inhibiting OCA2 expression.. Human Genetics.

[27] Abe K, Chisaka O, van Roy F, Takeichi M (2004). Stability of dendritic spines and synaptic contacts is controlled by alpha N-catenin.. Nature Neuroscience.

[28] Park C, Falls W, Finger JH, Longo-Guess CM, Ackerman SL (2002). Deletion in Catna2, encoding alpha N-catenin, causes cerebellar and hippocampal lamination defects and impaired startle modulation.. Nature Genetics.

[29] Branicki W, Brudnik U, Draus-Barini J, Kupiec T, Wojas-Pelc A (2008). Association of the SLC45A2 gene with physiological human hair colour variation.. J Hum Genet.

[30] Babor T, Higgins-Biddle JC, Saunders JB, Monteiro MG (2006). AUDIT: Alcohol Use Disorders Identification Test: guidelines for use in primary care..

[31] Bibikova M, Lin Z, Zhou L, Chudin E, Garcia EW (2006). High-throughput DNA methylation profiling using universal bead arrays.. Genome Res.

[32] Price AL, Patterson NJ, Plenge RM, Weinblatt ME, Shadick NA (2006). Principal components analysis corrects for stratification in genome-wide association studies.. Nat Genet.

[33] Purcell S, Neale B, Todd-Brown K, Thomas L, Ferreira MA (2007). PLINK: a tool set for whole-genome association and population-based linkage analyses.. Am J Hum Genet.

[34] Cardoso JF (1997). Infomax and maximum likelihood for blind source separation.. IEEE Signal Processing Letters.

[35] Huang da W, Sherman BT, Lempicki RA (2009). Systematic and integrative analysis of large gene lists using DAVID bioinformatics resources.. Nat Protoc.

[36] Dennis G, Sherman BT, Hosack DA, Yang J, Gao W (2003). DAVID: Database for Annotation, Visualization, and Integrated Discovery.. Genome Biol.

