# Supplementary material for: Identification of Genetic and Epigenetic Marks Involved in Population Structure
Source: PLoS One. 2010 Oct 7;5(10):e13209. doi: 10.1371/journal.pone.0013209 (PMC2951359; doi:10.1371/journal.pone.0013209)
Supplement: Table S1 — 8 SNPs in the 1st genetic PSF (0.05 MB DOC) [file pone.0013209.s001.doc]

Table S1. 8 SNPs in the 1^st^ genetic PSF

| **SNP** | **Chromosome** | **GENE** |
| --- | --- | --- |
| **rs16891982** | Chr.5 | SLC45A2: solute carrier family 45, member 2 |
| **rs35389** | Chr.5 | SLC45A2: solute carrier family 45, member 2 |
| **rs35407** | Chr.5 | SLC45A2: solute carrier family 45, member 2 |
| **rs35391** | Chr.5 | SLC45A2: solute carrier family 45, member 2 |
| **rs28117** | Chr.5 | SLC45A2: solute carrier family 45, member 2 |
| **rs3755095** | Chr.2 | CTNNA2: catenin (cadherin-associated protein), alpha 2 |
| **rs12913832** | Chr.15 | HERC2:hect domain and RLD 2 |
| **rs35412** | Chr.5 | SLC45A2: solute carrier family 45, member 2 |
