# Supplementary material for: Identification of Genetic and Epigenetic Marks Involved in Population Structure
Source: PLoS One. 2010 Oct 7;5(10):e13209. doi: 10.1371/journal.pone.0013209 (PMC2951359; doi:10.1371/journal.pone.0013209)
Supplement: Table S2 — Forty-eight sites in the methylation PSF (0.11 MB DOC) [file pone.0013209.s002.doc]

Table S2. Forty-eight sites in the methylation PSF

| **GENE symbol** | **Chromosome** | **Gene name** |
| --- | --- | --- |
| PM20D1 | Chr.1 | PM20D1 peptidase M20 domain containing 1 |
| DUSP22 | Chr.6 | dual specificity phosphatase 22 |
| TDG | Chr.12 | thymine-DNA glycosylase |
| PPP4R2 | Chr.3 | protein phosphatase 4, regulatory subunit 2 |
| ID2 | Chr.2 | inhibitor of DNA binding 2, dominant negative helix-loop-helix protein |
| DUSP22 | Chr.6 | dual specificity phosphatase 22 |
| DNAJB7 | Chr.22 | DnaJ (Hsp40) homolog, subfamily B, member 7 |
| PM20D1 | Chr.1 | PM20D1 peptidase M20 domain containing 1 |
| CAPN9 | Chr.1 | calpain 9 |
| CHST11 | Chr.12 | carbohydrate (chondroitin 4) sulfotransferase 11 |
| SULT1C2 | Chr.2 | sulfotransferase family, cytosolic, 1C, member 2 |
| LASS3 | Chr.15 | LAG1 homolog, ceramide synthase 3 |
| FAM71F1 | Chr.7 | family with sequence similarity 71, member F1 |
| C2orf40 | Chr.2 | chromosome 2 open reading frame 40 |
| TFAP2E | Chr.1 | transcription factor AP-2 epsilon (activating enhancer binding protein 2 epsilon) |
| SULT1C2 | Chr.2 | sulfotransferase family, cytosolic, 1C, member 2 |
| GSTM5 | Chr.1 | glutathione S-transferase mu 5 |
| NLRP5 | Chr.19 | NLR family, pyrin domain containing 5 |
| SLC44A4 | Chr.6 | solute carrier family 44, member 4 |
| SUN5 | Chr.20 | sperm associated antigen 4-like |
| SUSD1 | Chr.9 | sushi domain containing 1 |
| DEFA1 | Chr.8 | defensin, alpha 1 |
| CCL4 | Chr.17 | chemokine (C-C motif) ligand 4 |
| KRTCAP3 | Chr.2 | keratinocyte associated protein 3 |
| CCL26 | Chr.7 | chemokine (C-C motif) ligand 26 |
| GSTM1 | Chr.1 | glutathione S-transferase mu 1 |
| CDC42BPA | Chr.1 | CDC42 binding protein kinase alpha |
| IL6 | Chr.7 | interleukin 6 (interferon, beta 2) |
| HLA-DRA | Chr.6 | major histocompatibility complex, class II, DR alpha |
| CSDC2 | Chr.22 | cold shock domain containing C2, RNA binding |
| KLF17 | Chr.1 | Kruppel-like factor 17 |
| CCL4L2 | Chr.17 | chemokine (C-C motif) ligand 4-like 2 |
| GSTM5 | Chr.1 | glutathione S-transferase mu 5 |
| MRI1 | Chr.19 | methylthioribose-1-phosphate isomerase homolog |
| PSMD5 | Chr.9 | proteasome (prosome, macropain) 26S subunit, non-ATPase, 5. |
| STK38 | Chr.6 | serine/threonine kinase 38 |
| RHOJ | Chr.14 | ras homolog gene family, member J |
| CHFR | Chr.12 | checkpoint with forkhead and ring finger domains |
| UGT2B17 | Chr.4 | UDP glucuronosyltransferase 2 family, polypeptide B17 |
| RHD | Chr.1 | Rh blood group, D antigen |
| MRGPRX2 | Chr.11 | MAS-related GPR, member X2 |
| APOC2 | Chr.19 | apolipoprotein C-II |
| COL8A2 | Chr.1 | collagen, type VIII, alpha 2 |
| EPB41L1 | Chr.20 | erythrocyte membrane protein band 4.1-like 1 |
| FAM181A | Chr.14 | family with sequence similarity 181, member A |
| CYP2F1 | Chr.19 | cytochrome P450, family 2, subfamily F, polypeptide 1 |
| ZNF205 | Chr.16 | zinc finger protein 205 |
| SERPINB3 | Chr.18 | serpin peptidase inhibitor, clade B (ovalbumin), member 3 |
