# Supplementary material for: Identification of Genetic and Epigenetic Marks Involved in Population Structure
Source: PLoS One. 2010 Oct 7;5(10):e13209. doi: 10.1371/journal.pone.0013209 (PMC2951359; doi:10.1371/journal.pone.0013209)
Supplement: Table S3 — Biological process/annotation clusters (0.07 MB DOC) [file pone.0013209.s003.doc]

Table S3. Biological process/annotation clusters

| **Category** | **Term** | **Genes** |
| --- | --- | --- |
| **Annotation Cluster 1** | | |
| GO biological process KEGG_PATHWAY SP_PIR_KEYWORDS | taxis/ chemotaxis/ locomotory behavior/ behavior | IL6,DEFA1,CCL4L2,CCL4,CCL26,MRGPRX2 |
|  | inflammatory response/ defense response/ response to virus/ immune response/ response to wounding | IL6,DEFA1,CCL4L2,CCL4,HLA-DRA,CCL26 |
|  | *cell-cell signaling | IL6,CCL4L2,CCL4,CCL26,CTNNA2 |
|  | Cytokine-cytokine receptor interaction/ Chemokine signaling pathway / Cytosolic DNA-sensing pathway/ cytokine/ chemotaxis/ inflammatory response | IL6,CCL4L2,CCL4,CCL26 |
| **Annotation Cluster 2** | | |
| GO cellular component SP_PIR_KEYWORDS | extracellular region/ extracellular region part/ Secreted | C2ORF40,IL6,PM20D1,APOC2,DEFA1,CCL4L2,CCL4,COL8A2,CCL26 |
| **Annotation Cluster 3** | | |
| GO biological process | actin cytoskeleton organization/ actin filament-based process / cytoskeleton organization | RHOJ,EPB41L1,CDC42BPA |
| **Annotation Cluster 4** | | |
| GO biological process | *sexual reproduction/ gamete generation/ multicellular organism reproduction | NLRP5,SUN5,KLF17,HERC2 |
| **Annotation Cluster 5** | | |
| GO biological process | *cellular component morphogenesis/ cell morphogenesis/ cell adhesion/ | CCL4L2,CCL4,COL8A2,CTNNA2 |
| **Annotation Cluster 6** | | |
| GO biological process | *cellular macromolecule catabolic process/ cellular protein catabolic process | TDG,HERC2,PSMD5,CHFR |
| **Annotation Cluster 7** | | |
| SP_PIR_KEYWORDS GO molecular function | *zinc/zinc-finger | PM20D1,CDC42BPA,KLF17,HERC2,CHFR,ZNF205 |
|  | *transition metal ion binding/ zinc ion binding | CYP2F1,PM20D1,CDC42BPA,KLF17,HERC2,CHFR,ZNF205 |
| **Annotation Cluster 8** | | |
| GO biological process | intracellular signaling cascade/ phosphorus metabolic process | RHOJ,STK38,CDC42BPA,DUSP22 |
| **Annotation Cluster 9** | | |
| SP_PIR_KEYWORDS GO molecular function | *metal-binding | CYP2F1,STK38,PM20D1,CDC42BPA,KLF17,HERC2,CHFR,ZNF205 |
|  | *metal ion binding/ cation binding/ ion binding | CYP2F1,STK38,SUSD1,CAPN9,PM20D1,CDC42BPA,KLF17,HERC2,CHFR,ZNF205 |
| **Annotation Cluster 10** | | |
| UP_SEQ_FEATURE GO molecular function | nucleotide phosphate-binding region:ATP | NLRP5,STK38,CDC42BPA |
|  | nucleotide binding/adenyl nucleotide binding/ purine nucleotide binding/ nucleoside binding/ ATP binding/ ribonucleotide binding | RHOJ,NLRP5,STK38,CDC42BPA,CHFR,HLA-DRA |
| SP_PIR_KEYWORDS | nucleotide-binding/ ATP-binding | RHOJ,NLRP5,STK38,CDC42BPA |
| **Annotation Cluster 11** | | |
| GO cellular component | nuclear lumen/ nucleoplasm/ intracellular organelle lumen/ organelle lumen | NLRP5,STK38,TDG,CHFR |
| **Annotation Cluster 12** | | |
| UP_SEQ_FEATURE SP_PIR_KEYWORDS GO cellular component | *transmembrane region/ transmembrane | SLC45A2,UGT2B17,SLC44A4,LASS3,SUSD1,MRGPRX2,CHST11,RHD,HLA-DRA,KRTCAP3 |
|  | *integral to membrane/ intrinsic to membrane | SLC45A2,UGT2B17,IL6,SLC44A4,LASS3,SUSD1,MRGPRX2,CHST11,RHD,HLA-DRA,KRTCAP3 |
| **Annotation Cluster 13** | | |
| SP_PIR_KEYWORDS | DNA-binding/ transcription regulation | LASS3,KLF17,TFAP2E,ZNF205 |
| GO biological process | Transcription | KLF17,TFAP2E,ZNF205 |

Note: * highlights the biological processes where genes in SNP and methylation PSFs both involved.
